# Effects of fermented *Artemisia annua* on the intestinal microbiota and metabolites of Hu lambs with naturally infected with *Eimeria* spp.

**DOI:** 10.3389/fcimb.2024.1448516

**Published:** 2025-01-07

**Authors:** Shuaiqi Liu, Shiheng Li, Shunli Lu, Mingfan Yang, Manyu Liu, Juanfeng Li, Senyang Li, Fuchun Jian

**Affiliations:** ^1^ College of Veterinary Medicine, Henan Agricultural University, Zhengzhou, China; ^2^ International Joint Research Laboratory for Zoonotic Diseases of Henan, Zhengzhou, China; ^3^ Key Laboratory of Quality and Safety Control of Poultry Products (Zhengzhou), Ministry of Agriculture and Rural Affairs, Zhengzhou, China

**Keywords:** fermentation, *Artemisia annua*, *Eimeria*, Hu lambs, intestinal microbiota, metabolites

## Abstract

**Background:**

Sheep coccidiosis could disturb the balance of intestinal microbiota, causing diarrhea, and even death in lambs. Chemical drugs are the primary method of treating sheep coccidiosis, but their use will bring drug resistance, toxic side effects, drug residues, and other problems. Chinese herbal medicines are investigated as alternative methods for controlling coccidian infections.

**Methods:**

In this study, the effect of fermented *Artemisia annua* (FA) on oocysts per gram (OPG), average daily gain (ADG), and expression of inflammatory factors were investigated in lambs that were naturally infected with coccidia.

**Results:**

The results showed that the FA had similar anti-coccidiosis effect to the original drug, while the FA demonstrated a more significant effect on weight gain, and a better ability to reduce the inflammatory response compared to the unfermented drug during the treatment period (*P* < 0.05). Furthermore, High-throughput sequencing technology was used to study the effects of FA on intestinal microbiota, and fecal metabolites of naturally infected lambs. The species richness of intestinal microbiota of lambs was significantly improved by FA. The abundance of bacteria *unclassified_Muribaculaceae*, and *UCG_005* were increased by fermentation of *A. annua*. The abundance of bacteria *Escherichia_Shigella*, *unclassified_Clostridia_UCG_014*, and *Alistipes* was reduced. The prevention, and treatment of coccidiosis by fermentation of *A. annua* may also be related to a series of metabolites affected by intestinal microbiota, including artemisinin, Lysyl-Proline, and TRP-tyrosine.

**Conclusion:**

FA was found to have superior anti-coccidiosis, anti-inflammatory, and weight gain effects compared to the original *Artemisia annua*. Intestinal microbes and metabolites such as *unclassified_Muribaculaceae*, *UCG-005*, and Artemisinin were identified, suggesting their potential significance. *Alistipes* was proposed as a biomarker for predicting intestinal coccidia outbreak risk in lambs, pending further validation. The correlation between microbiota, and metabolites may provide new insights into pathogenic changes associated with *Eimeria* spp.

## Introduction

Coccidiosis is a common intestinal parasitic disease caused by parasites of the genus *Eimeria*. It mainly infects intestinal epithelial cells, causing intestinal damage, intestinal microbiota, and metabolic disorders (Campos et al., 2024). In addition, coccidian infection also affects the absorption of nutrients by the lamb’s gut, resulting in reduced feed conversion, diarrhea, and weight loss (Velazquez-González et al., 2022). These will cause the immune response of the lamb body, and susceptibility to intestinal pathogen infection, causing secondary infections, such as allergy, and inflammatory bowel disease (Morishima et al., 1984; Lu et al., 2021). In severe cases, it may cause bloody stools, or even death (Antonissen et al., 2016; Foreyt, 1990). With the development of the breeding industry, large-scale, and intensive sheep farms are becoming more mature, coccidiosis has caused serious economic losses to agricultural producers (Bangoura et al., 2022). At present, the treatment of coccidiosis is mainly based on chemical drugs, such as toltrazuril, albendazole, diclazuril (Akhmetzhanova et al., 2022; Le Sueur et al., 2009). However, discontinuing the drug is likely to cause a rebound of coccidian infection. Moreover, long-term usage may lead to issues such as drug resistance and drug residue (Odden et al., 2018; Bacanlı and Başaran, 2019). Therefore, it is of great significance to develop safe, green, and environmentally friendly new anticoccidial veterinary drugs.

*A. annua*, a Chinese herbal medicine, belongs to the genus *Artemisia* in the Asteraceae family, has anti-coccidiosis effect (Ghafouri et al., 2023). The main components include sesquiterpenoids, flavonoids, coumarins, triterpenoids, and phenolics (Xing et al., 2021; Yang et al., 2012). Artemisinin is a new sesquiterpene lactone compound with peroxy group isolated from the leaves of *A. annua*, which has effective anti-malaria properties (Paddon et al., 2013). In addition, *A. annua* also has good bactericidal, anti-inflammatory, immunity, and other effects (Meng et al., 2021; Nakase et al., 2008). However, for many Chinese herbs to function, they need to be converted into bioactive ingredients under the action of microorganisms in the host large intestine, or rumen (Lee et al., 2012). In animal production, Chinese herbs are used as feed additives, and the active ingredients, and active substances in the cells are difficult to pass through the cell wall. So it is difficult to be absorbed, and utilized by animals.

Probiotics have the ability to produce enzymes such as protease, cellulase, and galactosidase. They could induce the complex structure of cellulose in plant cell wall to decompose, and better release the active substances in Chinese herbal cells (Lv et al., 2019). Paddon found that the use of Saccharomyces cerevisiae yeast to fermented *Artemisia annua* can significantly increase the content of Artemisinin (Paddon et al., 2013). In addition, the use of probiotics to ferment Chinese herbs has the advantages of reducing some toxic components of Chinese herbs, improving the balance of intestinal microbiota, and micro-ecological environment, and improving the palatability of feed (Chen et al., 2020; Park et al., 2017; Xian-Tao et al., 2022). The use of *Bacillus subtilis* can regulate the intestinal microbiota, and maintain intestinal homeostasis, thereby alleviating intestinal disorders caused by *Eimeria* (Memon et al., 2022; Zou et al., 2022; Mi et al., 2022). This study aimed to evaluate the protective and growth-promoting effects of fermented *Artemisia annua* (FA) on coccidian infection lambs, as well as analyze the influence of FA treatment on intestinal microbiota, fecal metabolites, and inflammatory factors in lamb serum.

## Methods

### Preparation of experimental medicine

*A. annua* was purchased from Yuzhou Qianhe Pharmaceutical Co., Ltd. (Yuzhou, China), originating in Hubei, China. Probiotic liquid was provided by the Microbiology Laboratory of Henan Agricultural University. It contains *Bacillus subtilis*, *Bacillus licheniformis*, and *Lactobacillus plantarum*, with the concentration of each strain being ≥ 1.0×10^9^ CFU/mL. Dicazuril premix (0.5%) was obtained from Henan Hemu Animal Pharmaceutical Co., LTD. (Henan, China).

Fermented *Artemisia annua*:The *A. annua*, and the single steamed water (Water obtained by boiling ordinary water to vaporize it and condensing the resulting steam by distillation) were mixed thoroughly, and moistened according to the weight ratio of 10:3 w/v. Subsequently, 2.5% probiotic liquid was inoculated, and the mixture was placed in a one-way sealed fermentation bag. After being fully mixed with *A. annua*, air was discharged, and the sealing tape was sealed. The mixture was fermented at a humidity of 60% to 70%, and a temperature of 35°C to 37°C for 5 days.

### Animals, experimental design, and diets

**Figure 1:**
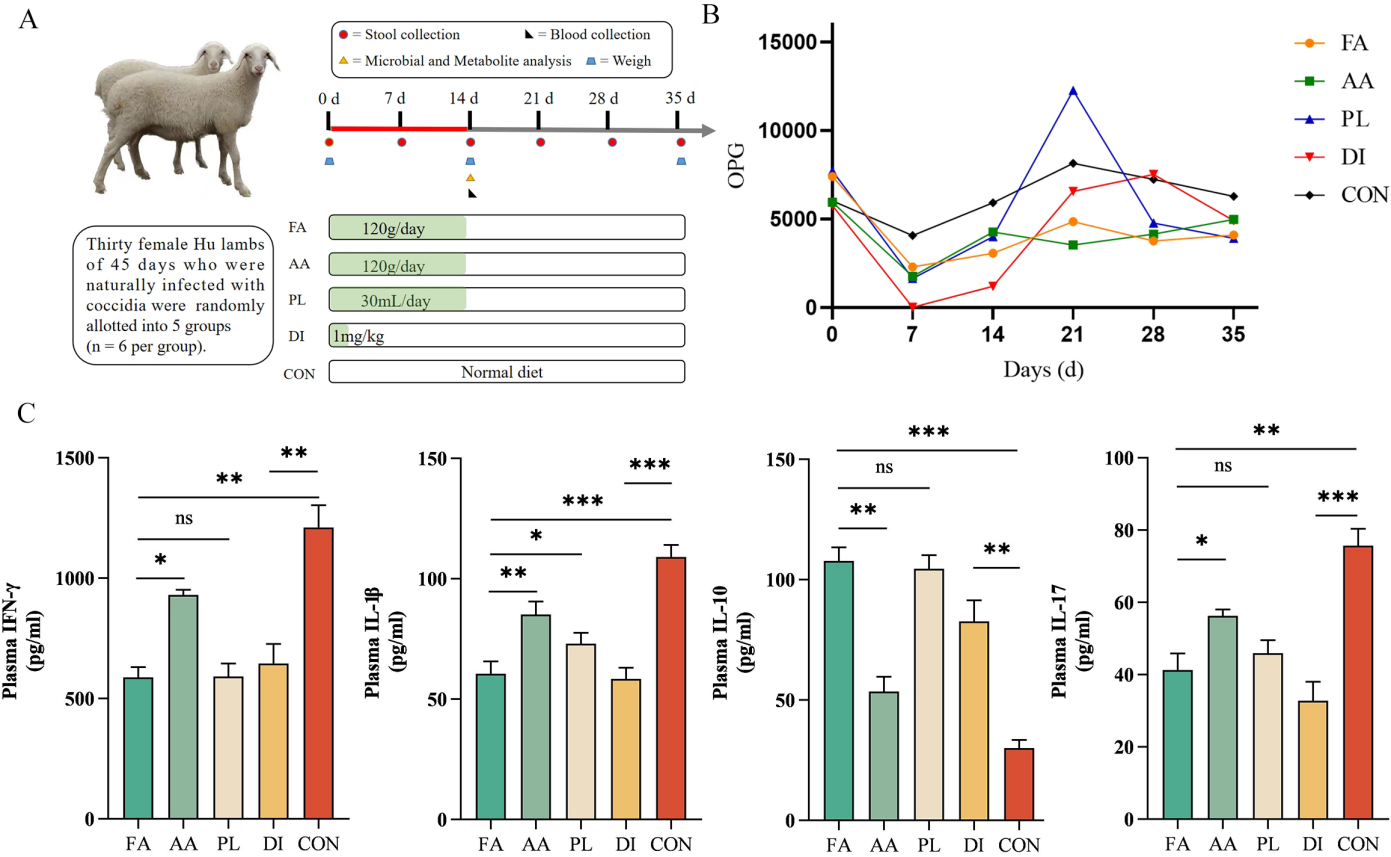
Effect of FA on OPG and serum inflammatory factors of naturally infected lambs. **(A)**. The flowchart of the test design. **(B)**. Changes in the number of oocysts per gram of feces (OPG) in lambs in each group on days 0, 7, 14, 21, 28, and 35, respectively (n = 6). Data are shown as means. **(C)**. Concentration of inflammatory factors (IFN-γ, IL-1β, IL-17 and IL-10) in plasma of lambs in different groups (n = 6). Data were presented as means ± SEM. *P < 0.05, **P < 0.01, ***P < 0.001. Fermented Artemisia annua = FA; Artemisia annua = AA; Probiotic liquid = PL; Diclazuril = DI; Control = CON. ns, Non - Significant.

Thirty 45-day-old female Hu sheep, naturally infected with coccidia, similar average weight, were provided by Henan Zhongyang Animal Husbandry Co., LTD. All experimental lambs had free access to feed, and water under standard conditions (concentrates + roughage). The primary formulations and ingredients of concentrate feed are shown in **Supplementary Table S1**. Additionally, in order to confirm that the amounts of coccidian infection in lambs were basically equal in each group, fecal samples were collected from the rectum to assess the number of oocysts per gram (OPG) by McMaster counting (Bauer et al., 2010; Bosco et al., 2014). Depending on the level of coccidian oocysts in the stool, lambs were divided into 5 equal groups with 6 replicates per group: (1) Fermented *Artemisia annua* (FA): Controlled addition of 120 g of fermented *Artemisia annua* to the diet; (2) *Artemisia annua* (AA): Controlled addition of 120 g of *Artemisia annua* to the diet; (3) Probiotic liquid (PL): Controlled addition of 30 mL of probiotic liquid to the diet; (4) Diclazuril (DI): Controlled addition of 1 mg/kg diclazuril to the diet, administered orally for 2 days; (5) Control group (CON): Normal diet. Different groups were fed separately in sheep house which with leakage dung floor. Under the same feeding conditions, the feeding time of the drug group was 14 days. The flowchart of the test design is shown in **Figure 1A**. The method of use and dosage of the drug refer to the Chinese Veterinary Pharmacopoeia.

### Data collection

#### Measurement of fecal oocyst enumeration

Rectal fecal samples were collected before morning feeding on days 0, 7, 14, 21, 28 and 35, respectively. Weigh 2g feces and thoroughly mix with 58mL saturated salt water. Filter the mixture through a mesh screen and collect the filtrate. The filtrate is driven into a McMaster counting plate and OPG counts are performed through an optical microscope. Subsequently, the relative reduction rate of oocysts was calculated. Relative reduction rate of oocysts = (number of oocysts in the medication group - number of oocysts in the control group)/number of oocysts in the control group ×100%.

#### Measurement of grow performance

Lambs were weighed on days 0, 14, and 35 of the experiment to calculate the average daily weight gain, and relative weight gain rate over the 14 days before the medication period, and the 35 days during the entire experiment. Average daily weight gain = (average final weight - average initial weight)/test days; Whereas the relative weight gain rate = (daily average weight gain of the experimental group - daily average weight gain of the control group)/daily average weight gain of the control group × 100%.

#### Determination of serum inflammatory factors

On the 14^th^ day of the experiment, blood was collected from the jugular vein of lambs in each group, centrifuged at 3000 × g at 4°C for 15 minutes to separate serum. IFN-γ, IL-1β, IL-17, and IL-10 were measured following the instructions provided in the kit. All the required kits were purchased from Shanghai Enzyme-Linked Biology Co., LTD.

Kit instructions: (1) Prepare all reagents before starting assay procedure. It is recommended that all Standards and Samples be added in duplicate to the Microelisa Stripplate. (2) Add standard: Set Standard wells, testing sample wells. Add standard 50 μl to standard well. (3) Add Sample: Add testing sample 10 μl then add Sample Diluent 40 μl to testing sample well; Blank well doesn’t add anyting. y 4) Add 100 μl of HRP-conjugate reagent to each well, cover with an adhesive strip and incubate for 60 minutes at 37°C. (5) Aspirate each well and wash, repeating the process four times for a total of five washes. Wash by filling each well with Wash Solution (400 μl) using a squirt bottle, manifold dispenser or autowasher. Complete removal of liquid at each step is essential to good performance. After the last wash, remove any remaining Wash Solution by aspirating or decanting. Invert the plate and blot it against clean paper towels. (6) Add chromogen solution A 50 μl and chromogen solution B 50 μl to each well. Gently mix and incubate for 15 minutes at 37°C. Protect from light. (7) Add 50 μl Stop Solution to each well. The color in the wells should change from blue to yellow. If the color in the wells is green or the color change does not appear uniform, gently tap the plate to ensure thorough mixing. (8) Read the Optical Density (O.D.) at 450 nm using a microtiter plate reader within 15 minutes.

#### 16S rRNA sequencing of intestinal microbiota

On the 14^th^ days, approximately 1 g of fresh stool collected from the rectum was placed in 2 mL EP tube, immediately placed in dry ice, and sent to Biomeiker Technologies (BMK) for sequecing. Total genomic DNA was extracted from stool samples using the TGuide S96 Magnetic Stool DNA Kit (Tiangen Biotech (Beijing) Co., Ltd.) according to manufacturer’s instructions. The hypervariable region V3-V4 of the bacterial 16S rRNA gene were amplified with primer pairs 338F: 5’- ACTCCTACGGGAGGCAGCA-3’ and 806R: 5’- GGACTACHVGGGTWTCTAAT-3’. After with initial denaturation at 95°C for 5 min, followed by 20 cycles of denaturation at 95°C for 30 s, annealing at 50°C for 30 s, and extension at 72°C for 40 s, and a final step at 72°C for 7 min. PCR products were checked on agarose gel and purified through the Omega DNA purification kit (Omega Inc., Norcross, GA, USA). The purified PCR products were collected and the paired ends (2 × 250 bp) was performed on the Illumina Novaseq 6000 platform.

#### Metabolomic profiling of fecal samples

The LC/MS system for metabolomics analysis is composed of Waters Acquity I-Class PLUS ultra-high performance liquid tandem Waters Xevo G2-XS QTof high resolution mass spectrometer. The column used is purchased from Waters Acquity UPLC HSS T3 column (1.8um 2.1*100mm). Waters Xevo G2-XS QTOF high resolution mass spectrometer can collect primary and secondary mass spectrometry data in MSe mode under the control of the acquisition software (MassLynx V4.2, Waters). In each data acquisition cycle, dual-channel data acquisition can be performed on both low collision energy and high collision energy at the same time. The low collision energy is 2 V, the high collision energy range is 10~40V, and the scanning frequency is 0.2 seconds for a mass spectrum. The parameters of the ESI ion source are as follows: Capillary voltage: 2000 V (positive ion mode) or -1500V (negative ion mode); cone voltage: 30 V; ion source temperature: 150°C; desolvent gas temperature 500°C; backflush gas flow rate: 50 L/h; Desolventizing gas flow rate: 800 L/h.

The raw data collected using MassLynx V4.2 is processed by Progenesis QI software for peak extraction, peak alignment and other data processing operations, based on the Progenesis QI software online METLIN database and Biomark’s self-built library for identification. At the same time, theoretical fragment identification and mass deviation All are within 100ppm.

#### Bioinformatic and statistical analysis

Taxonomy annotation of the OTUs/ASVs was performed based on the Naive Bayes classifier in QIIME2 using the SILVA database (release 138.1) with a confidence threshold of 70% (Bolyen et al., 2019; Quast et al., 2013). Alpha was performed to identify the complexity of species diversity of each sample utilizing QIIME2 software. Beta diversity calculations were analyzed by principal coordinate analysis (PCoA) to assess the diversity in samples for species complexity. One-way analysis of variance was used to compare bacterial abundance and diversity. Linear discriminant analysis (LDA) coupled with effect size (LEfSe) was applied to evaluate the differentially abundant taxa. The online platform BMKCloud (https://www.biocloud.net) was used to analyze the sequencing data.

Principal component analysis and Spearman correlation analysis were used to judge the repeatability of the samples within group and the quality control samples. The identified compounds are searched for classification and pathway information in KEGG, HMDB and lipidmaps databases. According to the grouping information, calculate and compare the difference multiples, T test was used to calculate the difference significance p value of each compound. The R language package ropls was used to perform OPLS-DA modeling, and 200 times permutation tests was performed to verify the reliability of the model. The VIP value of the model was calculated using multiple cross-validation. The method of combining the difference multiple, the P value and the VIP value of the OPLS-DA model was adopted to screen the differential metabolites. The screening criteria are FC>1, P value<0.01 and VIP>1. The difference metabolites of KEGG pathway enrichment significance were calculated using hypergeometric distribution test.

One-way analysis of variance (ANOVA) was performed for the data following the normal-distribution and expressed as mean ± standard deviation (SD) using GraphPad Prism 8.0.2 (GraphPad Software, La Jolla, CA, USA). Employ Duncan test to compare and demonstrate statistically significant differences in daily weight gain and inflammatory factors among distinct treatment groups. Non-parametric test (Kruskal-Wallis test) was conducted for data that did not conform to normal-distribution. The threshold of statistical significance was set at *P* ≤ 0.05.

## Results

### Effect of FA on OPG of naturally infected lambs

According to the line chart of coccidia output, and OPG before, and after different treatment groups (**Figure 1B**; **Table 1**). At the beginning of treatment, all groups had a good anti-coccidiosis effect on lambs. The OPG of FA group at 7, 14, 21, 28, and 35 days was lower than that of the CON group, but there was no significant difference (*P* > 0.05). Compared with AA, and PL groups, the relative reduction rate of coccidian oocysts in FA group was more stable (**Table 2**). It was worth noting that the relative reduction rate of coccidian oocysts in PL group was -50.51% after discontinuation (21 d). In the DI group, the anti-coccidiosis effect was the most obvious on day 7 (*P* < 0.05), with the relative reduction rate of coccidian oocysts was 99.58%. However, on day 21 after drug withdrawal, the relative reduction rate of coccidian oocysts was 19.51%. The above results showed that the FA group had similar anti-coccidiosis effects as the DI group, and these effects were stable, and long-lasting after 2 weeks of administration. The DI group can almost completely inhibit the production of coccidian oocysts in the intestinal tract of lambs during the early stages of administration. However, upon discontinuation, it is easy to cause rapid rebound of the number of coccidian infections in the intestinal tract of lambs, and the efficacy is not long-lasting, which is easy to cause stress reaction in lambs.

**Table 1:** OPG values before and after different medication groups.

Groups	Time (Day)					
	0d	7 d	14 d	21 d	28 d	35 d
FA	4200 (2500~13350)	1800 (900~3300)	2500 (950~4800)	4800 (3800~5700)	2600 (1175~7575)	3750 (1225~5875)
AA	4900 (3125~9550)	1000 (725~3475)	3300 (1825~7275)	3600 (2150~4550)	3700 (1600~6375)	5050 (4150~5900)
PL	2300 (875~13725)	900 (550~2550)	3400 (925~7725)	12200 (11950~12750)	1700 (1225~8500)	3350 (500~6700)
DI	4850 (1950~10075)	0* (0~25)	450 (175~2800)	5300 (2250~20850)	7600 (4550~9700)	3050 (2125~8475)
CON	2700 (1975~8625)	1950 (1175~6400)	6250 (1775~10200)	5700 (2000~15600)	6150 (1800~11000)	5300 (3550~10075)

OPG means oocysts per gram of feces; Statistical analysis was performed by Kruskal-Willis test. * indicates a significant difference (*P* < 0.05) compared with the CON group.

Fermented *Artemisia annua =* FA; *Artemisia annua* = AA; Probiotic liquid = PL; Diclazuril = DI; Control = CON.

**Table 2:** Relative reduction rate of coccidian oocysts in each group at different periods compared with the control group.

Groups	Relative reduction rate^a^				
	7d	14d	21d	28d	35d
FA	43.45%	48.31%	40.34%	48.04%	34.71%
AA	56.55%	28.08%	56.60%	42.76%	20.78%
PL	59.68%	32.58%	-50.51%	34.03%	37.63%
DI	99.58%	79.77%	19.51%	-3.72%	21.70%
CON	/	/	/	/	/

a Relative reduction rate of oocysts = (number of oocysts in the medication group - number of oocysts in the control group)/number of oocysts in the control group ×100%

Fermented *Artemisia annua =* FA; *Artemisia annua* = AA; Probiotic liquid = PL; Diclazuril = DI; Control = CON.

### Effect of FA on weight gain of lambs

The results in **Table 3** showed that there was no significant difference in the body weight of lambs at the 14d, and 35d trial stages (*P* > 0.05). In order to observe the weight change between the groups more clearly. We calculated the average daily weight gain, and relative weight gain rate of the groups during the medication period (0 to 14d), and the non-medication period (14 to 35d). For the AA group, the effect of FA group on promoting lamb weight gain was enhanced. Compared with the CON group, the average daily weight gain in the FA group during the treatment period was 134g (*P* < 0.05). However, the relative weight gain rate during the non-treatment period was 29.75%. It showed no significant difference compared to the CON group (*P* > 0.05), and the weight gain effect was similar to that in the AA group. The weight gain effect of the PL group was more stable, and the relative weight gain rate was more than 70% observed in both the medication period, and the non-medication period. There was a significant difference compared to the CON group during the non-medication period (*P* < 0.05). Compared with CON group, ADG in DI group was not significantly improved. The above results showed that the FA had a significant improvement in the weight gain of lambs. However, the weight gain effect after discontinuation of the drug was not as good as in the PL group. This may be due to the probiotics in the PL group had colonized in the intestine, and could continue to play a role.

**Table 3:** Effects of drugs on growth performance of lambs.

Groups	Initial BW (kg)	Final BW (kg)	ADG (g)^A^	rADG (%)^B^	SEM	*P*-value
0-14 d						
FA	15.3 ± 2.15	17.17 ± 2.43	134 ± 28^B^	108.52	11	0.031
AA	18.02 ± 3.04	19.13 ± 2.84	80 ± 42^AB^	24.63	17	0.621
PL	15.63 ± 1.8	17.32 ± 2.66	120 ± 99^AB^	87.87	40	0.077
DI	17.13 ± 2.57	18.03 ± 2.94	77 ± 31^AB^	1.38	12	0.683
CON	18.1 ± 4.02	19 ± 4.07	64 ± 21^A^	–	9	–
14-35 d						
FA	17.17 ± 2.43	20.68 ± 3.12	167 ± 105^ab^	29.75	25	0.113
AA	19.13 ± 2.84	22.33 ± 2.64	153 ± 18^ab^	18.12	8	0.514
PL	17.32 ± 2.66	21.93 ± 3.35	220 ± 127^b^	70.42	25	0.02
DI	18.03 ± 2.94	21.2 ± 2.65	117 ± 47^ab^	16.89	19	0.667
CON	19 ± 4.07	21.7 ± 4.13	103 ± 65^a^	–	27	–

^A^ ADG (g), Daily average weight gain=(average final weight - average initial weight)/test days

^B^ rADG (%), Relative daily average weight gain rate=(daily average weight gain of the experimental group - daily average weight gain of the control group)/daily average weight gain of the control group × 100%

Different capital letters indicated that ADG of different groups at 14d was significantly different (*P* < 0.05). Different lowercase letters indicated significant difference in ADG at 35d (*P* < 0.05).

SEM and P-value describe ADG indicators, adoption makes a Least Significant Difference (LSD).

Fermented *Artemisia annua =* FA; *Artemisia annua* = AA; Probiotic liquid = PL; Diclazuril = DI; Control = CON.

### Effect of FA on serum inflammatory factors of lambs

When treating lambs naturally infected with coccidia, each medication group had a great influence on the changes in anti-inflammatory, and pro-inflammatory factors in the serum of the lambs. **Figure 1C**; **Table 4** showed the expression levels of four inflammatory factors in each group on the 14 d of the experiment. The expression of pro-inflammatory factors (IFN-γ, IL-1β, IL-17) was significantly decreased. The expression level of anti-inflammatory factor IL-10 was significantly increased (*P* < 0.01) in the FA group compared to the CON, and AA groups. However, there were no significant differences in IFN-γ, IL-17, and IL-10 expression levels between the FA, and PL groups (*P* > 0.05). In addition, compared with CON group, the pro-inflammatory factors (IFN-γ, IL-1β, IL-17) were significantly decreased in DI group, and the anti-inflammatory factors (IL-10) were significantly increased. These results suggested that the expression of pro-inflammatory factors in the serum of lambs could be significantly reduced, and an anti-inflammatory factor was induced after using FA. The effect was more significant than that of the *A. annua* drug.

**Table 4:** Expressions of inflammatory factors in serum of lambs in each group.

Groups	IFN-γ (pg/mL)	IL-1β (pg/mL)	IL-10 (pg/mL)	IL-17 (pg/mL)
FA	587.56 ± 43.49^a^	60.61 ± 5.05^a^	107.84 ± 5.54^d^	41.28 ± 4.62^b^
AA	930.94 ± 20.87^b^	85.21 ± 5.39^c^	53.62 ± 6.02^b^	56.31 ± 1.76^c^
PL	591.58 ± 54.03^a^	73.06 ± 4.54^b^	104.57 ± 5.67^d^	45.96 ± 3.6^b^
DI	645.44 ± 81.99^a^	58.43 ± 4.6^a^	82.71 ± 8.77^c^	32.81 ± 5.22^a^
CON	1210.4 ± 92.12^c^	109.01 ± 5.03^d^	30.06 ± 3.35^a^	75.7 ± 4.71^d^

There was significant difference in the representation of the same column data without the same letter label (*P* < 0.05).

Fermented *Artemisia annua =* FA; *Artemisia annua* = AA; Probiotic liquid = PL; Diclazuril = DI; Control = CON.

### Effect of FA on intestinal microbiota of lambs

#### Effect of FA on intestinal microbiota diversity of lambs naturally infected with coccidia

A total of 3065243 original sequences were obtained from 30 fecal samples of Hu sheep (FA=699343, AA=460808, PL=960114, DI=468783, CON=476195), and at least 460808 sequences were generated from each sample, with an average of 613049 sequences (**Supplementary Table S2**).

**Figure 2:**
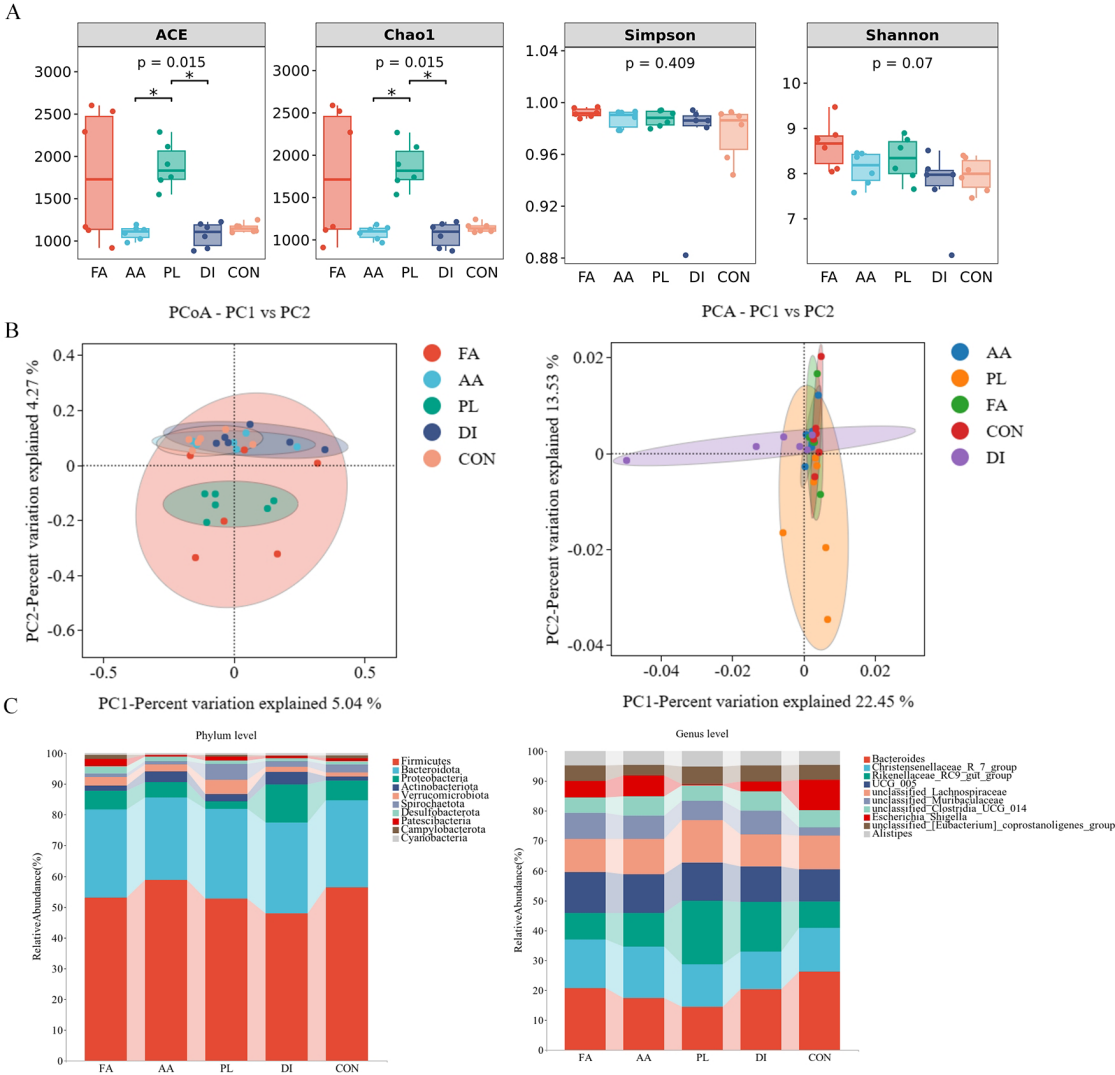
Effect of FA on intestinal microbiota of lambs naturally infected with coccidia. **(A)**. Alpha diversity as presented by ACE, chao1, shannon and simpson indexs in the intestinal contents of sheep among groups. Among them, the first two represent the richness of microbial community, and the last two represent the diversity of microbial community (n = 6). **(B)**. Principal coordinates analysis (PCoA) and Principal component analysis (PCA) of bacterial community in sheep intestinal contents (n = 6). **(C)**. Histogram analysis of gut bacterial taxonomic composition at phylum and genus level (n = 6). *Fermented Artemisia annua* = FA; *Artemisia annua* = AA; Probiotic liquid = PL; Diclazuril = DI; Control = CON. (*P < 0.05).

As can be seen in **Figure 2A**, the ACE index, and Chao1 index were increased by feeding FA, and PL for 14 consecutive days. But there was no significant difference compared with CON group (*P* > 0.05). The ACE index, and Chao1 index of AA, and DI groups were decreased to a certain extent compared with CON group. The highest Simpson index, and Shannon index were in the FA group, which had no significant difference compared with CON group (*P* > 0.05). Therefore, we can conclude that feeding *A. annua* to lambs after fermentation can improve the diversity, and richness of intestinal microorganisms.

The PCoA map showed that FA, and PL groups were separated from the CON group microbiota structure to a certain extent. The PCA map showed structural similarity of the gut microbes between the groups (**Figure 2B**). The relative dispersion among individuals within the FA group can be explained by the effect of individual differences among lambs within the FA group. Therefore, the results showed that the structure of the *A. annua* flora was changed by fermentation to some extent.

#### Effect of FA on intestinal microbiota composition of lambs naturally infected with coccidia

**Figure 3:**
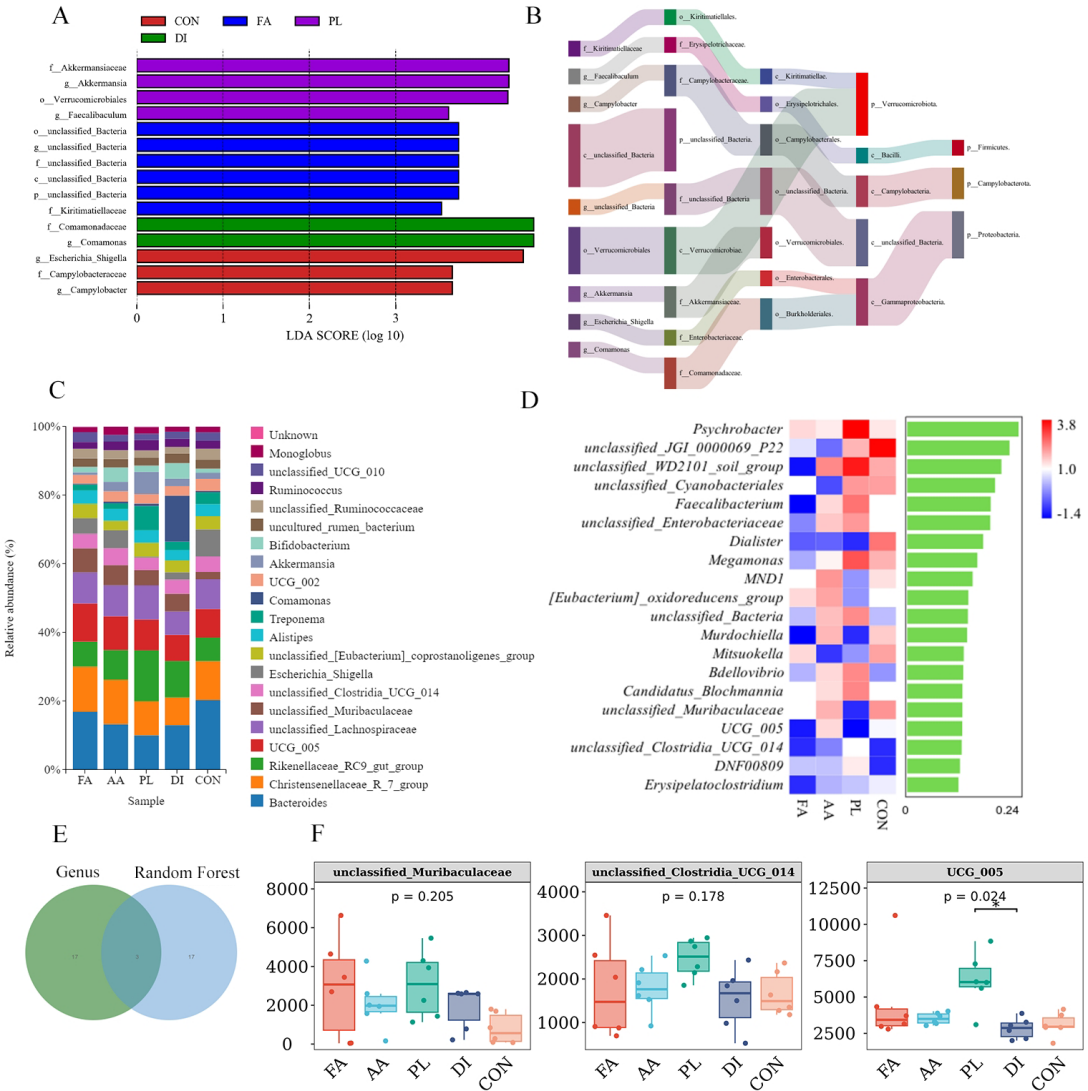
Screening for landmark differences. **(A)**. LEfSe (Line Discriminant Analysis (LDA) Effect Size) analysis showing the most significantly abundant taxa of the intestinal microbial community in each group (LEfSe LDA>3.5, P<0.05) (n = 6). **(B)**. The dominant taxa on intestinal microbiota from phylum to genus were analyzed using Sankey diagram (LDA>3.5) (n = 6). **(C)**. The top 20 genera of bacteria with abundances (n = 6). **(D)**. The top 20 bacterial genera were distributed according to their importance (n = 6). **(E)**. Venn diagrams for each group of the top 20 species in abundance and the top 20 in importance. **(F)**. Difference in the abundances of intestinal microbiota (including *unclassified Muribaculaceae*, *unclassified ClostridiaUCG 014*, and *UCG_005*) at the genus level (n = 6). *Fermented Artemisia annua* = FA; *Artemisia annua* = AA; Probiotic liquid = PL; Diclazuril = DI; Control = CON. (*P < 0.05).

As shown in the histogram of species distribution. At the phylum level, Firmicutes (51.17%), Bacteroidota (28.46%), and Verrucomicrobiota (4.65%) were the predominant bacteria in the PL group. The dominant bacteria in FA, AA, DI, and CON groups were Firmicutes (50.02%, 58.63%, 47.77%, and 56.24%), Bacteroidota (26.99%, 26.70%, 29.53%, 28.15%), and Proteobacteria (5.62%, 4.93%, 12.29%, 6.49%), respectively (**Figure 3A**; **Supplementary Table S3**). At the genus level, *Bacteroides* (9.31%, 9.93%, 6.98%, 9.29%, 14.70%), *Christensenellaceae R-7 group* (7.21%, 9.66%, 6.89%, 5.80%, 8.20%), *Rikenellaceae RC9_gut_group* (4.02%, 6.47%, 10.30%, 7.60%, 4.96%), UCG 005 (6.08%, 7.32%, 6.21%, 5.43%, 6.01%), and *unclassified Lachnospiraceae* (4.98%, 6.74%, 6.90%, 4.90%, 6.24%) was the dominant genus of FA, AA, PL, DI, and CON groups. Accounting for more than 30% of the bacterial composition (**Figure 2C**; **Supplementary Table S4**).

#### Effect of FA on intestinal differential bacterial analysis of lambs naturally infected with coccidia

Fifteen different bacteria were selected by LEfSe (Line Discriminant Analysis (LDA) Effect Size) analysis (LEfSe LDA>3.5, *P*<0.05). These were FA group (o_unclassified_Bacteria, g_*unclassified_Bacteria*, f_unclassified_Bacteria, c_unclassified_Bacteria, p_unclassified_Bacteria), PL group (f_Akkermansiaceae,g_*Akkermansia*,o_Verrucomicrobiales,g_*Faecalibaculum*), DI group (f_Comamonadaceae,g_*Comamonas*), CON group (g_*Escherichia_Shigella*,f_Campylobacteraceae,g_*Campylobacter*). No different bacteria were found in group AA under these screening conditions (**Figure 3A**). The analysis of Sankey chart showed that the intestinal microbiota of CON group mainly belonged to Campylobacterota, and Proteobacteria. The intestinal microbiota of FA, and PL groups mainly belonged to Firmicutes, and Verrucomicrobiota (**Figure 3B**). These results showed that FA, and PL could change the dominant groups of lambs naturally infected with coccidia. In addition, we made a random forest map of the top 20 important bacterial genus according to their importance classification (**Figures 3C, D**). *Psychrobacter*, *unclassified_JGI_0000069_P22*, and *unclassified_WD2101_soil_group* were the top 3 most important bacteria. Venn analysis was performed on the top 20 important bacteria genus and the top 20 abundance ratio. Three difference bacteria were finally screened out, which were *unclassified_Muribaculaceae*, *unclassified_Clostridia_UCG_014*, and *UCG_005*, respectively (**Figure 3E**). As shown in **Figure 3F**, compared with the CON group, the abundance of *unclassified_Muribaculaceae* in the FA, and PL groups increased (*P* > 0.05). The abundance of *unclassified_Clostridia_UCG_014*, and *UCG_005* in PL group was higher than that in other groups. And *UCG_005* in the PL group was significantly different from that in the DI group (*P* < 0.05).

#### Correlation analysis of intestinal microbiota with inflammatory factors, OPG, and ADG

**Figure 4:**
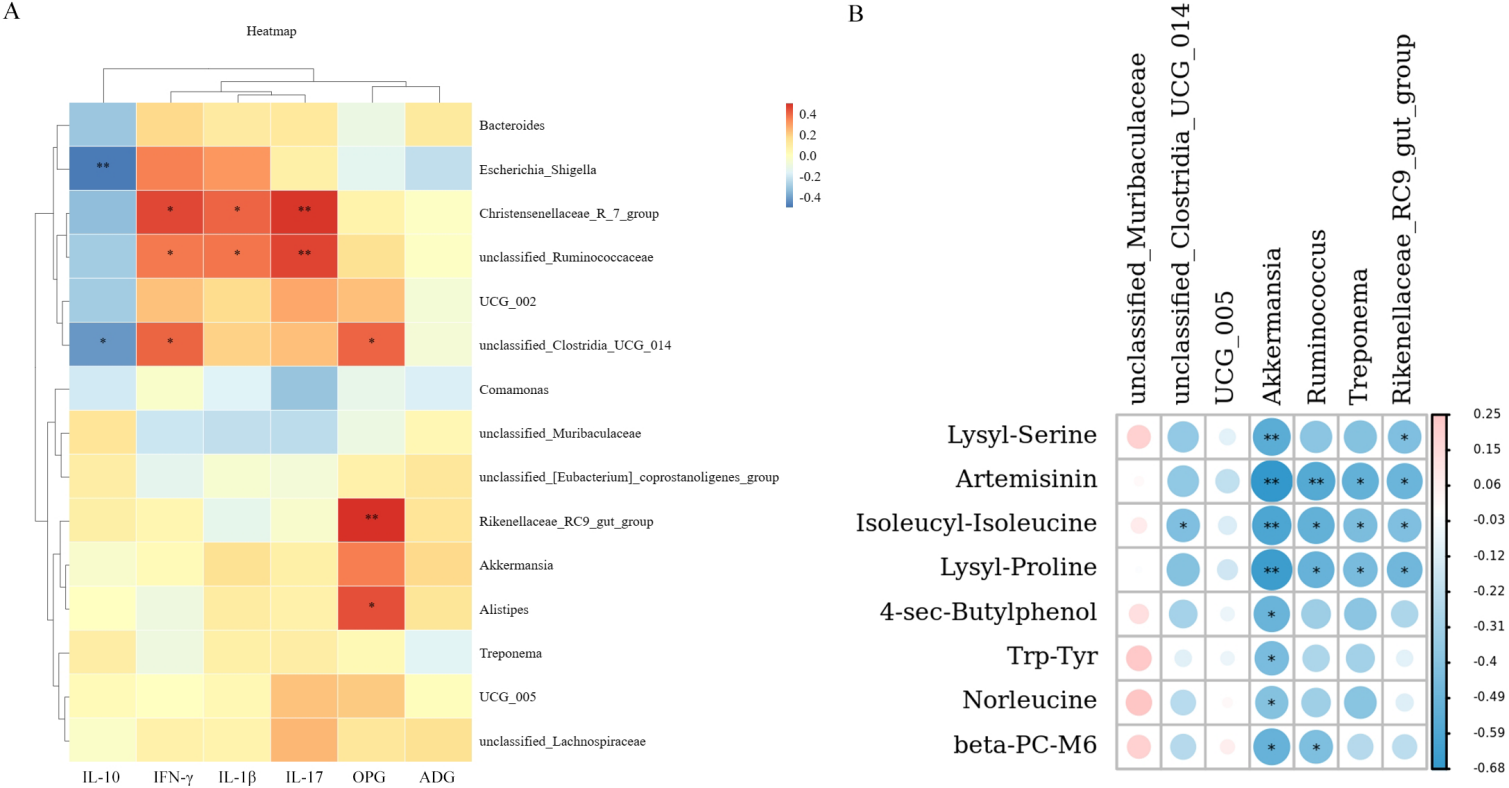
Heat map of correlation analysis. **(A)**. Spearman correlation analysis of intestinal microbiota with inflammatory factors, OPG and ADG. **(B)**. Spearman correlation coefficient was used to analyze the correlation between differential metabolites, and intestinal microbiota. * *P* < 0.05, * * *P* < 0.01. Red represents positive correlation, blue represents negative correlation (n = 6). Fermented *Artemisia annua =* FA; *Artemisia annua* = AA; Probiotic liquid = PL; Diclazuril = DI; Control = CON.

In order to understand the relationship between inflammatory factors, weight gain effect, coccidian infection amount, and intestinal microbiota of lambs. Spearman correlation analysis was used to investigate the correlation between IL-10, IFN-γ, IL-1β, IL-17, OPG, ADG, and genus level flora of lambs at 14 days (**Figure 4A**). The results showed that *Escherichia_Shigella*, and *unclassified_Clostridia_UCG_014* were significantly negatively correlated with IL-10 (*P* < 0.05). *Christensenellaceae_R_7_group*, and *unclassified_Ruminococcaceae* were significantly positively correlated with IFN-γ, IL-1β, and IL-17 (*P* < 0.05). *Unclassified_Clostridia_UCG_014* was significantly positively correlated with IFN-γ (*P* < 0.05). *Rikenellaceae_RC9_gut_group* was significantly positively correlated with OPG (*P* < 0.01). And *unclassified_Clostridia_UCG_014*, and *Alistipes* were significantly positively correlated with OPG (*P* < 0.05). *Escherichia_Shigella* was positively correlated with ADG, but there was no significant difference (*P* > 0.05).

### Effect of FA on metabolites in lamb feces

**Figure 5:**
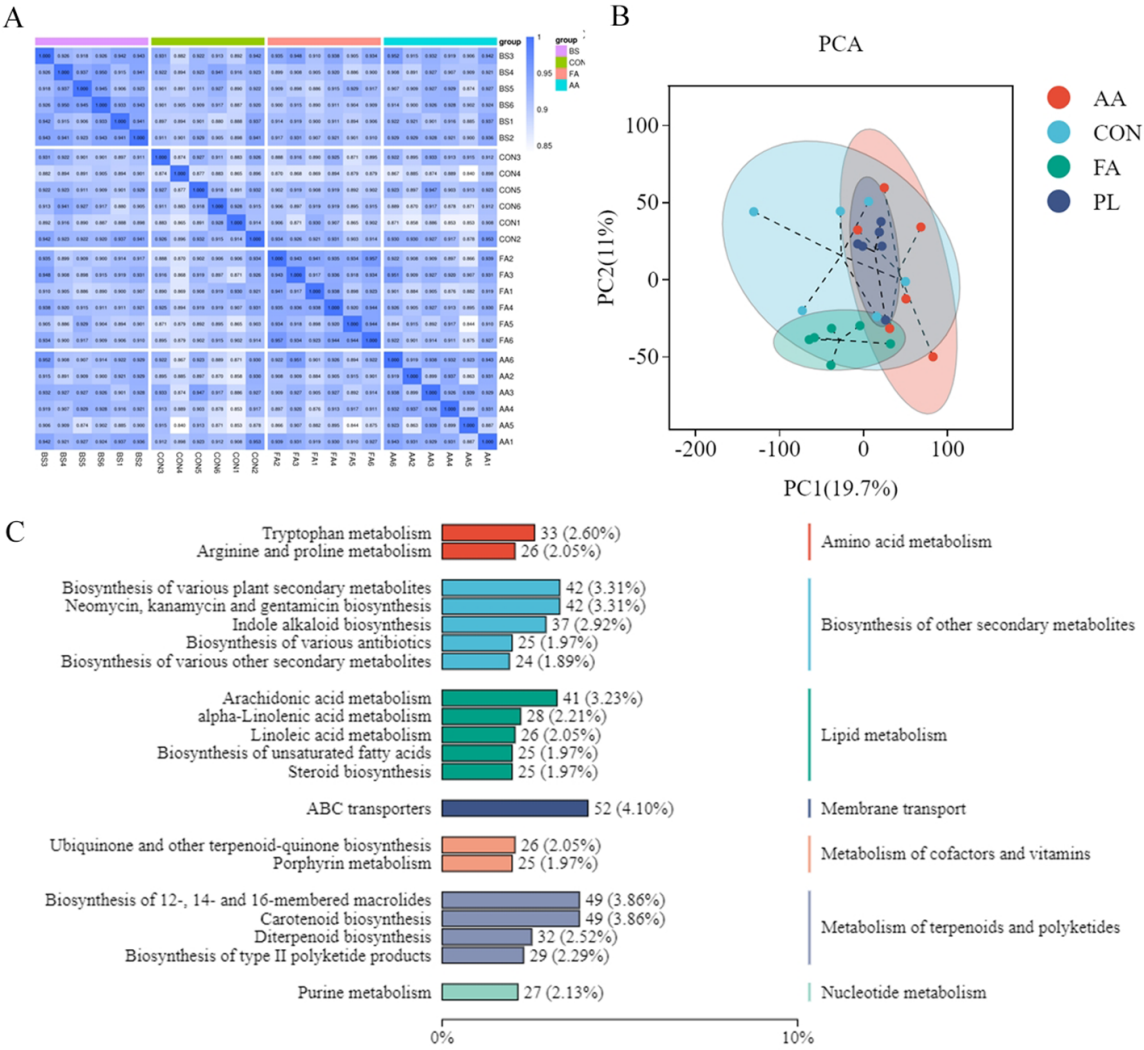
Effect of FA on metabolites in lamb feces. **(A)**. Data quality assessment. Biological duplication between samples within a group can be assessed by correlation analysis between samples. At the same time, the higher the correlation coefficient between intra-group samples and inter-group samples, the more reliable the differential metabolites obtained. Spearman Rank Correlation (r) was used as an evaluation index of biological repeat correlation. The closer r is to 1, the stronger the correlation between the two duplicate samples. **(B)**. PCA analysis. Through principal component analysis of samples (including quality control samples), we can preliminarily understand the overall metabolic differences between samples and the variation degree between samples in the group. **(C)**. Functional annotation of metabolite KEGG. The KEGG database was used to annotate all identified metabolites, and the top20 annotated information with the most annotations in KO pathway level2 entries were selected. Fermented *Artemisia annua =* FA; *Artemisia annua* = AA; Probiotic liquid = PL; Diclazuril = DI; Control = CON.

The data on sample repeatability within the group, and sample differences between the groups were evaluated (**Figures 5A, B**). There was a strong correlation between the two repeated samples, and there was a certain distance in metabolite structure, and different degrees of overlap between the drug groups. It shows that the data quality evaluation is satisfactory, and the metabolite data analysis can be carried out.

Qualitative, and quantitative metabolomic analysis was performed on 30 fecal samples, and a total of 3,010 metabolites were annotated in default mode. Major fecal metabolites annotated using the KEGG database include Metabolism of terpenoids and polyketides, Nucleotide metabolism, and Biosynthesis of other secondary metabolites, etc. Metabolism of terpenoids, and polyketides is the most abundant metabolite, followed by Metabolism of terpenoids, and polyketides (**Figure 5C**).

#### Differential metabolite screening

**Figure 6:**
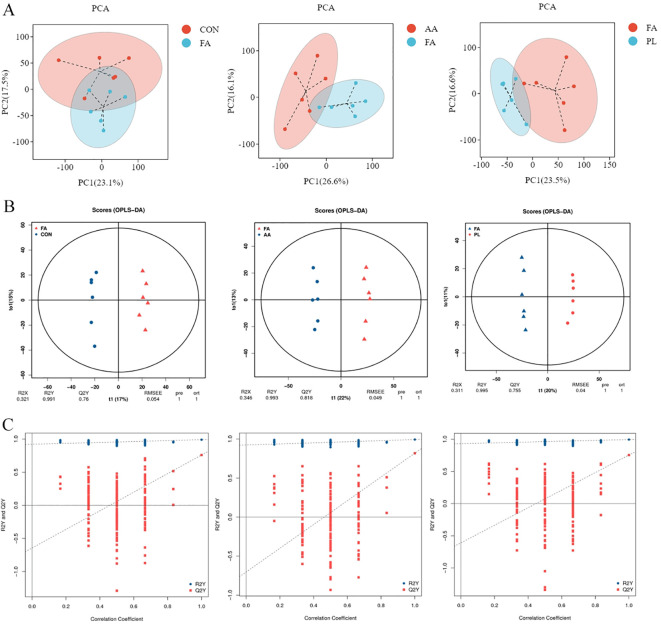
Multivariate statistical analysis of intestinal metabolism. **(A)**. Grouped principal component analysis. **(B)**. OPLS-DA score chart. Generally speaking, Q2Y>0.5 can be regarded as an effective model, and Q2Y>0.9 is an excellent model. **(C)**. OPLS-DA model replacement test diagram. If the slope of the regression line fitted by Q2Y is regular, it means that the model is meaningful, and the blue dot is generally above the red dot, it means that the independence of the training set and the test set of the modeling is good. The figures in **(B, C)** represent respectively FA vs CON, FA vs AA, FA vs PL. Fermented *Artemisia annua =* FA; *Artemisia annua* = AA; Probiotic liquid = PL; Diclazuril = DI; Control = CON.

The identified metabolites were evaluated using orthogonal partial least squares discriminant analysis (OPLS-DA), and PCA (**Figure 6**). The results showed that samples from the same group gathered together, indicating that the variation of samples within the group was small. There were different degrees of separation between samples from different groups. However, there was also a small amount of overlap, indicating that intestinal metabolites changed to a certain extent between the different groups (**Figure 6A**). If the slope of the regression line fitted by Q2Y was regular. It means that the model was meaningful, and the blue dot was generally above the red dot. It means that the independence between the training set, and the test set of the model was good. Therefore, the overall results indicate that these data were stable, and reliable, and can be used for differential metabolite analysis (**Figures 6B, C**).

**Figure 7:**
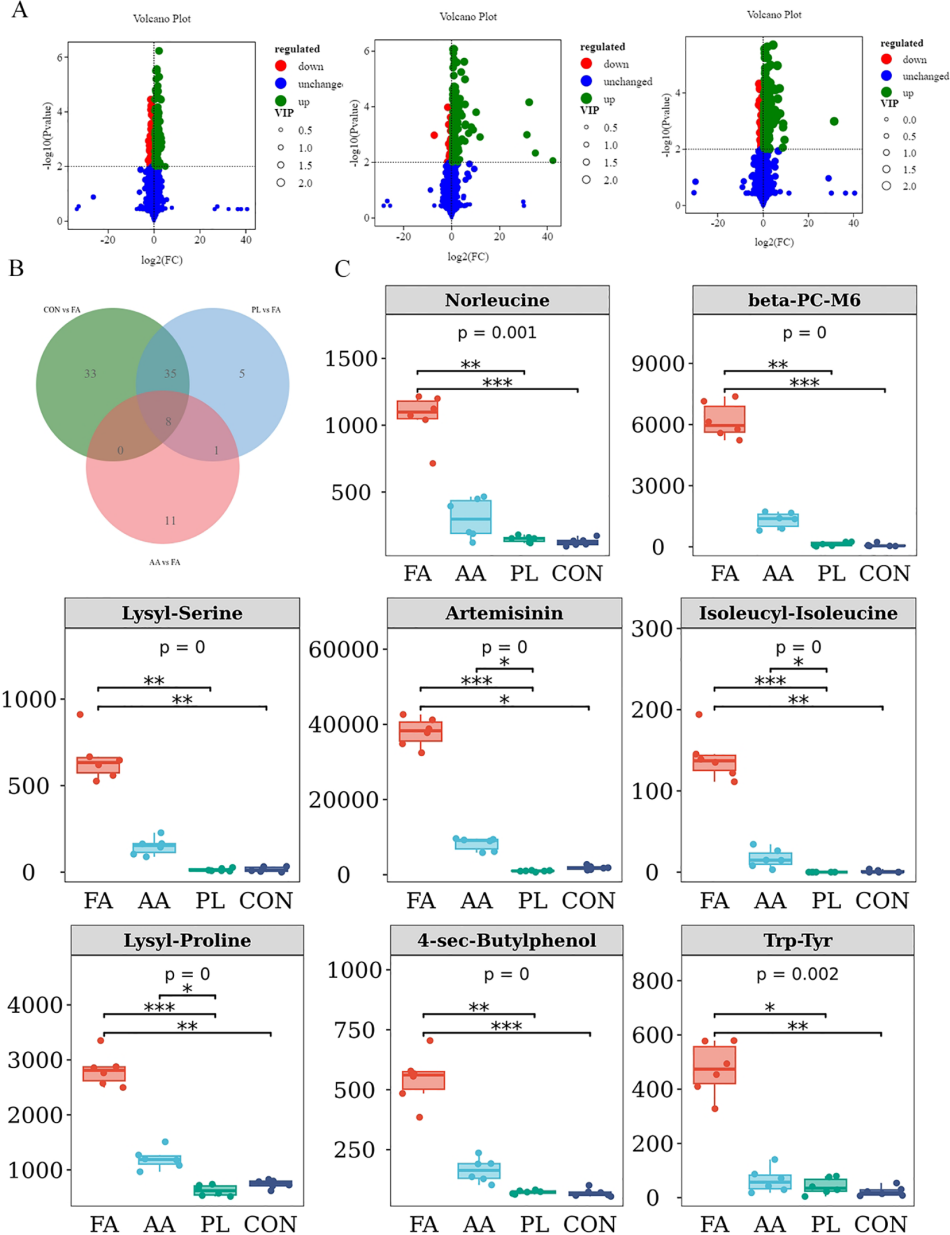
Marker differential metabolite screening. **(A)**. Volcano plot of significant differentially expressed metabolite of FA vs CON, FA vs AA, FA vs PL. Red dots, downregulated; green dots, upregulated. **(B)**. Venn shows differential metabolites shared by FA vs CON, FA vs AA, FA vs PL. **(C)**. The screened differential metabolites were represented by box diagram. * *P* < 0.05, * * *P* < 0.01, *** *P* < 0.001. Fermented *Artemisia annua =* FA; *Artemisia annua* = AA; Probiotic liquid = PL; Diclazuril = DI; Control = CON.

The differential metabolites between the FA group, and the CON, AA, and PL groups. They were analyzed according to the criteria of FC=1, P value=0.01, and VIP=1, and visualized by the volcano map (**Figure 7A**). The number of different metabolites in CON, and FA groups was 210, including 168 up-regulated metabolites, and 42 down-regulated metabolites. The number of different metabolites in AA, and FA groups was 307, including 163 up-regulated metabolites, and 144 down-regulated metabolites. The number of different metabolites in PL, and FA groups was 277, including 219 up-regulated metabolites, and 58 down-regulated metabolites (**Table 5**). In order to further screen the key metabolites between groups as potential biomarkers. VIP value >2, and |log2 (FC) | ≥ 1 were selected as screening criteria. Venn diagram was used to analyze the common differential metabolites in FA group, and CON, AA and PL groups that met screening criteria. As shown in **Figures 7B, C**, a total of 8 core differential metabolites were identified by Venn diagram analysis. They include Artemisinin, beta-PC-M6, Lysyl-Proline, Trp-Tyr, Isoleucyl-Isoleucine, Norleucine, 4-sec-Butylphenol, Lysyl-Serine. According to the box pattern, all 8 selected species in the FA group were significantly up-regulated (*P* < 0.001).

**Table 5:** Statistical table of differential metabolites.

Group	Diff-num	Up-num	Down-num
AA_vs_FA	307	163	144
PL_vs_FA	277	219	58
CON_vs_FA	210	168	42

Group: differential metabolite grouping information;

Diff-num: number of metabolites with significant difference; Up-num: number of up-regulated metabolites; Down-num: number of down-regulated metabolites.

Fermented *Artemisia annua =* FA; *Artemisia annua* = AA; Probiotic liquid = PL; Diclazuril = DI; Control = CON.

#### Correlation analysis between differential metabolites, and intestinal microbiota

Spearman correlation coefficient was used to analyze the correlation between differential metabolites, and intestinal microbiota (**Figure 4B**). The results showed that *unclassified_Clostridia_UCG_014* was significantly negatively correlated with Isoleucyl-Isoleucine (r > 0.4, *P* < 0.05). *Akkermansia* was associated with Lysyl-Serine, Artemisinin, Isoleucyl-Isoleucine, Lysyl-Proline, 4-sec-Butylphenol, Trp-Tyr, Norleucine, and beta-PC-M6, there was a significant negative correlation (r > 0.4, *P* < 0.05). *Ruminococcus* was significantly negatively correlated with Artemisinin, Isoleucyl-Isoleucine, Lysyl-Proline, and beta-PC-M (r > 0.4, *P* < 0.05). *Treponema* was negatively correlated with Artemisinin, Isoleucyl-Isoleucine, and Lysyl-Proline (r > 0.4, *P* < 0.05). *Rikenellaceae_RC9_gut_group* was significantly negatively correlated with Lysyl-Serine, Artemisinin, Isoleucyl-Isoleucine, and Lysyl-Proline (r > 0.4, *P* < 0.05). In addition, *unclassified_Muribaculaceae* was positively correlated with Lysyl-Serine, Isoleucyl-Isoleucine, 4-sec-Butylphenol, Trp-Tyr, Norleucine, and beta-PC-M6, while there was no significant difference (*P* < 0.05). These results indicated that the changes of metabolites in lamb feces were closely related to intestinal microbiota.

## Discussion

Coccidian infection causes serious clinical symptoms in weaned lambs, the most common of which are watery diarrhea, and bloody stool (Velazquez-González et al., 2022). In addition, coccidian infection can also cause intestinal cell destruction, and intestinal damage to different degrees, causing intestinal microflora disorders (Tomal et al., 2023; Wang et al., 2021). Previous studies have shown that *A. annua* not only has an anti-coccidiosis effect, but also affects the intestinal microbial environment, and intestinal inflammation (He et al., 2022). Probiotics have beneficial immunomodulatory effects on acute infectious diarrhea, antibiotic-associated diarrhea, hepatic encephalopathy, ulcerative colitis, and necrotizing enterocolitis (Kim et al., 2019; Wilkins and Sequoia, 2017). Therefore, we used probiotics to ferment *A. annua* to explore whether FA could significantly regulate intestinal microbiota, and metabolism. So as to reduce inflammatory response, and weight loss caused by coccidian infection. Our results showed that the anti-coccididial effect, promoting weight gain, and inhibiting inflammation caused by coccidian infection of lambs were enhanced to varying degrees after fermentation of *A. annua*. And we speculate that fermentation of *A. annua* may be achieved by improving the intestinal microbiota, and increasing beneficial metabolites in lambs.

The results of our experiment showed that FA had similar anti-coccidiosis effect as the original drug of *A. annua*. And the relative reduction rate of coccidian oocysts in FA group was generally higher in the whole experiment cycle. Artemisinin, an active component of *A. annua*, promotes the apoptosis of the second generation schizosomal cells of coccidia by inhibiting the expression of transcription factor NF-κB, and anti-apoptotic factor Bcl-xL (Lorea Baroja et al., 2007; Kim et al., 2019). In addition, it has been found that Artemisinin inhibits the expression of SERCA in the endoplasmic reticulum of coccidian macrogametes. Resulting in the imbalance of Ca^2+^ homeostasis in coccidian cells, thus inhibiting the formation, and sporification of the oocyst wall of coccidian (del Cacho et al., 2010). In the screening results of fecal differential metabolites in this study (**Figure 7**). Artemisinin composition in group FA was significantly higher than that in the group AA, which was also consistent with our results of anti-coccidiosis. Therefore, after the probiotic fermentation of *A. annua*, the active components of anti-coccidiosis in *A. annua* could be absorbed more easily by the intestinal tract of lambs. Du Huijie et al. explored the anti-coccidiosis infection effect of compound probiotics. And the results showed that probiotics could promote chick weight gain, reduce cecal lesions, and reduce the output of coccidian oocysts, thus playing an anti-coccidiosis effect, which was consistent with our research results (Du et al., 2019). PL group had anti-coccidiosis effect during the treatment period, but the coccidian infection amount rebounded after discontinuation. In terms of weight gain, the relative weight gain rate of the PL group was increased during both the medication, and non-medication periods. These result indicated that feeding probiotics to lambs for 14 consecutive days could successfully colonize the intestinal tract of lambs, and play a role. However, the weight gain effect of FA was not significantly different from the original *A. annua* in the non-treatment period. We speculated that the significant increase in lamb weight gain in the FA group during the medication period could be due to the role of probiotics. But there was no significant difference between the FA group, and the AA group during the non-medication period. This may be due to the fact that the dose of probiotics used in fermentation was too small, and did not colonize the lamb intestine. In addition, studies have reported that IL-1β participates in inflammation, and tissue damage, and is positively proportional to intestinal mucosal barrier function indicators, and opportunistic pathogens, and inversely proportional to dominant bacteria (Feng et al., 2023). IL-10 is an important immunomodulator, which mainly inhibits, and terminates the inflammatory immune response by inhibiting the activation of monocytes, and macrophages (Bouquet et al., 2020). IFN-γ plays an important role in the prevention, and control of coccidiosis. On days 4, and 8 after coccidian infection. The transcription levels of IFN-γ immune-related genes in the spleen of Tibetan chickens were significantly upregulated, and coccidian infection triggered a strong T cell immune response driven by IFN-γ (Yun et al., 2000; Li et al., 2021). Zhang Lei et al. showed that chicken IL-17A played a role in aggravating pathological injury in the process of coccidian infection (Zhang et al., 2011). These results are consistent with our study. In this study, the results of serum inflammatory factors on the 14^th^ day of each group showed that the expression of pro-inflammatory factors (IFN-γ, IL-1β, IL-17). And anti-inflammatory factor (IL-10) in serum of lambs were decreased by FA, *A. annua* drug, and probiotic solution. But after fermentation, *A. annua* had the most remarkable effect. The results showed that after fermentation, *A. annua* could better reduce the inflammatory response caused by coccidian infection.

The intestinal microbiota intricately participates in the biosynthesis of essential vitamins, notably vitamin K and various B vitamins critical for maintaining normal physiological functions in animals. Disruptions in the structural balance of the intestinal microbiota can precipitate inadequate vitamin synthesis, thereby exerting detrimental effects on animal health (Lee et al., 2022). A thriving gut microbiome is pivotal in upholding the integrity of the intestinal epithelium, which serves as a formidable physical barrier against the infiltration of deleterious agents such as bacteria, viruses, and toxins. Through intricate interactions with intestinal epithelial cells, the microbiota facilitates the upregulation of tight junction proteins, thereby bolstering the impermeability of the intestinal barrier. Furthermore, the gut microbiome assumes a central role in fostering the development and maturation of the immune system, contributing significantly to the formation of gut-associated lymphoid tissue (GALT) and the enhancement of differentiation and functional maturation of immune cells such as T cells, B cells, and macrophages, crucial components in the defense against pathogenic invasions (Kayama et al., 2020; Gao et al., 2024). Consequently, the configuration and functionality of the gut microbiota wield profound effects on animal health across multifaceted dimensions.

Plant extracts have emerged as potent agents in modulating the composition of the gut microbiota, with a plethora of studies substantiating their favorable impact on the ecological equilibrium of the gut microbiota. Plant extracts, including Broussonetia papyrifera leaf extract, Xiasangju residue, and Danggui Shaoyao San, exhibit distinctive capabilities in influencing the intestinal microbiota, thereby offering promising avenues for sustaining intestinal health and enhancing associated physiological functions (Liang et al., 2023; Sun et al., 2023; Liu et al., 2022). Hence, within this study, we embark on an investigation into the repercussions of Artemisia annua on the intestinal flora of lambs afflicted with natural coccidial infestations.

Increased Alpha diversity in microbiota analysis is thought to be closely related to homeostasis, and health in the animal intestinal. High diversity of intestinal microbiota can reduce the host’s sensitivity to pathogenic parasites such as *Eimeria*, improve intestinal immunity, and promote food digestion, and absorption (Huang et al., 2018; Li et al., 2023). In this study, both FA, and PL significantly improved the diversity of intestinal microbiota in lambs. The dominant bacterial at phylum, and genus level were further analyzed. Firmicutes, and Bacteroidota were the main bacterial phylum. At genus level, *Bacteroides*, *Christensenellaceae_R_7_group*, *Rikenellaceae_RC9_gut_group*, *UCG_005*, and *unclassified_Lachnospiraceae* are the main bacteroides genus. In addition, a total of 15 different microorganisms were identified by intergroup difference analysis (LEfSe analysis). It is worth noting that the significant dominant genus screened in the CON group are *Escherichia_Shigella*, and *Campylobacter*. Among them, *Escherichia_Shigella* is closely related to a variety of diseases of the digestive tract, and is an opportunistic pathogen of the intestine, whose abundance is too high will lead to microecological disorders (Zhao et al., 2017). *Campylobacter* infection can cause enteritis, bacteremia, hepatitis, pancreatitis, meningitis, suppurative arthritis, and other diseases (Awofisayo-Okuyelu et al., 2017). Therefore, these results suggested that coccidian infection increases the abundance of pathogenic bacteria such as *Escherichia_Shigella*, and *Campylobacter*, which will increased the risk of disease in the host. However, the dominant bacteria of naturally infected lambs with coccidia were changed by feeding with FA, effectively reducing the abundance of harmful bacteria. Three difference bacteria, *unclassified_Muribaculaceae*, *unclassified_Clostridia_UCG_014*, and *UCG_005*, were screened by Venn analysis. The abundance of *unclassified_Muribaculaceae* was higher in FA, and PL groups. Increasing the abundance of *unclassified_Muribaculaceae* plays an important role in improving inflammation, and intestinal structure (He et al., 2021; Yu et al., 2021). *Unclassified_Muribaculaceae* has been reported to be positively associated with butyrate, which improves intestinal barrier function. Butyric acid can improve intestinal barrier function (Wang et al., 2023). Therefore, FA may improve intestinal injury, and inflammation caused by coccidian-infected lambs by increasing the abundance of *unclassified_Muribaculaceae*, and promote better intestinal absorption of nutrients. *Clostridia_UCG-014* is positively correlated with blood glucose concentration, and is increased in diabetic, and obese mice (Liu et al., 2023). Whereas studies on coccidiosis, and inflammation have not been reported. In our study, *Clostridia_UCG-014* showed a significant positive correlation with OPG, and pro-inflammatory factor, and a significant negative correlation with anti-inflammatory factor, so we could conclude that *Clostridia_UCG-014* is harmful bacteria in this study. In addition, in this study, the abundance of *UCG-005* in the PL group was significantly higher than that in the DI group, and the PL group had the best weight gain effect. Relevant studies have shown that *UCG_005* is positively correlated with host growth, and body weight, and the abundance of *UCG-005* in the intestinal tract of diarrhea goats is significantly decreased (Tang et al., 2020; Wang et al., 2018). This is consistent with our findings. Studies have shown that *Alistipes* may be harmful, or beneficial. In this study, the correlation analysis showed that *Alistipes* was significantly positively correlated with OPG. It has been reported that *Alistipes* is pathogenic in rectal cancer, and the imbalance of intestinal microbiota may be related to the abundance of *Alistipes* (Parker et al., 2020). So *Alistipes* is a harmful bacterium in our study. However, in the research results of Chen Pan’s study on *Radix Dichroae* for the treatment of sheep coccidiosis, *Alistipes* was a beneficial bacterium (Chen et al., 2024). Which was inconsistent with our research result, and further research was still needed. Above that, the differential markers *unclassified_Muribaculaceae*, and *UCG-005* were screened to play an important role in host defense against coccidian damage.

Lamb gut microbiota can also play a mediating role *in vivo* through the production of multiple metabolites. We performed fecal metabolomics analysis. The FA group was compared with the PL group, AA group, and CON group, and the differential metabolites were screened. The Venn analysis was performed. The results showed that, 8 species of Artemisinin, beta-PC-M6, Lysyl-Proline, Trp-Tyr, Isoleucyl-Isoleucine, Norleucine, 4-sec-Butylphenol, Lysyl-Serine were significantly up-regulated by fermentation of Artemisinin Heterometabolites. Lysyl-Proline, TRP-tyrosine, Isoleucyl-Isoleucine, Norleucine, and Lysyl-Serine are common amino acids in animals. They play different roles in supplementing nutrition, improving metabolism, promoting intestinal digestion, and improving immunity. Trp-Tyr metabolites could regulate mucosal barrier immunity. They can activate the expression of inflammatory factors such as IL-10, IL-17, and IL-22 after binding with aromatic hydrocarbon receptors, and also regulate the expression of tight junction protein (Bowerman et al., 2020; Singh et al., 2019). Studies have shown that dietary supplementation of Isoleucyl-Isoleucine can improve the growth performance of broilers, improve the meat color of broilers, increase the protein content in muscle, and improve the development of intestinal villi (Liu et al., 2024). Many studies have reported that gut microbes drive phenotypic characteristics of animals, and the level of metabolites in the gut may affect the function of gut microbiota (Liu et al., 2019; Morgavi et al., 2015). In this study, we did not carry out high performance liquid chromatography (HPLC) analysis of the fermented *A. annua*, which is also the work we need to study in the future. However, after differential metabolite screening, Artemisinin composition in the FA group was significantly higher than that in the AA group. Artemisinin, which is mainly produced by fermented *A. annua*, can reduce the output of coccididian oocysts by inhibiting the formation of the oocysts wall, and can also reduce the sporification rate of coccididian oocysts, showing certain anti-coccidiosis effect (Jiao, 2016). Therefore, we speculated that the increase in the content of these beneficial metabolites may be one of the ways that FA exerts its beneficial effects. The effect of active ingredients in FA on pharmacological mechanisms such as intestinal flora, and metabolites needs further study. In this study, we found a close relationship between intestinal microbes, and differential metabolites in lambs through correlation analysis. It is conducive to further understanding the function of intestinal microbes, and their metabolites. At the same time, it also suggests that the anti-coccidiosis, and growth-promoting effects of FA may be realized through the interaction between intestinal microorganisms, and metabolites.

## Conclusion

In our study, the reduction rate of coccidium oocysts of fermented *Artemisia annua* remained at 30% - 50% during the whole test cycle, and the efficacy was more lasting than that of Dicazuril. During the treatment period, fermented *Artemisia annua* could significantly increase the weight gain of lambs (*P* < 0.05), and the average daily weight gain was 134 g. In addition, We found that the anticoccidial and growth-promoting effects of fermented *Artemisia annua* were related to intestinal flora (*unclassified_Muribaculaceae*, *UCG_005*, *Alistipes*, *unclassified_Clostridia_UCG_014* and *Escherichia_Shigella*), inflammatory factors (IFN-γ, IL-1β, IL-17, IL-10), and metabolites (Artemisinin, Lysyl-Proline, Trp-Tyr), which needs our further verification in the later stage. From the perspective of intestinal flora and metabolism, the theoretical basis was provided for the mechanism of Chinese herbal medicine to prevent coccidiosis.

## Data Availability

The data presented in the study are deposited in the NCBI repository, accession number PRJNA1197987.
