# Supplementary material for: Effects of fermented Artemisia annua on the intestinal microbiota and metabolites of Hu lambs with naturally infected with Eimeria spp
Source: Front Cell Infect Microbiol. 2025 Jan 7;14:1448516. doi: 10.3389/fcimb.2024.1448516 (PMC11747653; doi:10.3389/fcimb.2024.1448516)
Supplement: Supplementary file 1 [file Table1.docx]

Table S1 Guaranteed Composition Analysis (%)

| Crude protein | Crude fiber | Crude ash | Sodium chloride |
| --- | --- | --- | --- |
| ≥18.5 | ≤15.0 | ≤10.0 | 0.40-1.50 |
| Calcium | Total phosphorus | Lysine | Water content |
| 0.6-2.0 | ≥0.3 | ≥0.40 | ≤14.0 |

Raw material composition: corn, soybean meal, extruded soybean, extruded corn, dicalcium phosphate, stone powder, sodium chloride, amino acids, composite vitamins, composite trace elements, choline chloride, enzyme preparations, etc.

Table S2 Statistical analysis of sequencing sequences

| Sample ID | Raw Reads | Clean Reads | Denoised Reads | Merged Reads | Non-chimeric Reads |
| --- | --- | --- | --- | --- | --- |
| FA1 | 79976 | 71723 | 71119 | 57612 | 49056 |
| FA2 | 79842 | 72044 | 71643 | 61643 | 52157 |
| FA3 | 79959 | 71864 | 71364 | 59142 | 50399 |
| FA4 | 160045 | 141204 | 139962 | 121282 | 109709 |
| FA5 | 139570 | 123748 | 122382 | 102382 | 95035 |
| FA6 | 159951 | 138196 | 137158 | 119925 | 101934 |
| AA1 | 80169 | 71842 | 71361 | 58429 | 47480 |
| AA2 | 80129 | 72021 | 71620 | 60932 | 49547 |
| AA3 | 80068 | 72343 | 71749 | 59294 | 50461 |
| AA4 | 60563 | 54266 | 53892 | 46381 | 41425 |
| AA5 | 79986 | 72357 | 71951 | 61303 | 53737 |
| AA6 | 79893 | 71636 | 71035 | 59193 | 48385 |
| PL1 | 159898 | 137019 | 135901 | 113406 | 90683 |
| PL2 | 159974 | 138939 | 137955 | 119384 | 102461 |
| PL3 | 160242 | 139678 | 138728 | 123474 | 105146 |
| PL4 | 160032 | 137803 | 136959 | 118625 | 102940 |
| PL5 | 159924 | 137699 | 136863 | 118932 | 102044 |
| PL6 | 160044 | 137172 | 135939 | 116948 | 97241 |
| DI1 | 79995 | 71527 | 71035 | 61687 | 52940 |
| DI2 | 80005 | 71486 | 71068 | 62951 | 56301 |
| DI3 | 79771 | 71166 | 70649 | 60700 | 53212 |
| DI4 | 68844 | 61664 | 61349 | 55193 | 48323 |
| DI5 | 80102 | 71580 | 71018 | 58206 | 47963 |
| DI6 | 80066 | 72086 | 71724 | 64670 | 57862 |
| CON1 | 79929 | 72234 | 71789 | 61307 | 54130 |
| CON2 | 80013 | 71481 | 71080 | 62827 | 55775 |
| CON3 | 79950 | 72012 | 71522 | 61448 | 51892 |
| CON4 | 79898 | 71342 | 70789 | 61004 | 51971 |
| CON5 | 79832 | 71481 | 71040 | 58644 | 50706 |
| CON6 | 76573 | 68723 | 68271 | 56186 | 46706 |

Table S3 The proportion of bacteria in each group was top 10 at phylum level

| phylum | FA | AA | PL | DI | CON |
| --- | --- | --- | --- | --- | --- |
| Firmicutes | 0.500245947 | 0.586304863 | 0.51166152 | 0.477734394 | 0.56240196 |
| Bacteroidota | 0.269875968 | 0.267035769 | 0.284635431 | 0.295293799 | 0.281460259 |
| Proteobacteria | 0.056221586 | 0.049329933 | 0.022590247 | 0.122861651 | 0.064891038 |
| Actinobacteriota | 0.016616338 | 0.035498981 | 0.022650892 | 0.04075414 | 0.012241207 |
| Verrucomicrobiota | 0.025686612 | 0.021387391 | 0.046518053 | 0.016466126 | 0.013748266 |
| Spirochaetota | 0.010153326 | 0.011741758 | 0.049403742 | 0.017317163 | 0.024816236 |
| Desulfobacterota | 0.023696975 | 0.013425586 | 0.009869967 | 0.010626803 | 0.012153701 |
| Patescibacteria | 0.022497017 | 0.005501892 | 0.012652897 | 0.006371619 | 0.008731218 |
| Campylobacterota | 0.011240788 | 0.001181452 | 0.00649238 | 0.002919661 | 0.009962793 |
| Cyanobacteria | 0.005598336 | 0.00474313 | 0.005060485 | 0.006722234 | 0.006446323 |
| Others | 0.05761345 | 0.003835387 | 0.027591772 | 0.002906912 | 0.003124311 |
| Unknown | 0.000553657 | 0.000013859 | 0.000872614 | 0.000025499 | 0.000022687 |

Table S4 The proportion of bacteria in each group was top 10 at genus level

| genus | FA | AA | PL | DI | CON |
| --- | --- | --- | --- | --- | --- |
| Bacteroides | 0.093120311 | 0.099335477 | 0.06979561 | 0.092944683 | 0.146965788 |
| Christensenellaceae_R_7_group | 0.072090156 | 0.096615713 | 0.068917942 | 0.057966118 | 0.08200993 |
| Rikenellaceae_RC9_gut_group | 0.040174347 | 0.064695733 | 0.103022309 | 0.075974947 | 0.049616267 |
| UCG_005 | 0.060844956 | 0.073187633 | 0.062137499 | 0.054303791 | 0.060133269 |
| unclassified_Lachnospiraceae | 0.049822543 | 0.067394709 | 0.069015648 | 0.04900314 | 0.062414924 |
| unclassified_Muribaculaceae | 0.038623665 | 0.043540474 | 0.031183301 | 0.036489394 | 0.015530809 |
| unclassified_Clostridia_UCG_014 | 0.023218315 | 0.03717588 | 0.024997515 | 0.029480294 | 0.032335325 |
| Escherichia_Shigella | 0.025007224 | 0.039136882 | 0.001829456 | 0.015149728 | 0.057125634 |
| unclassified_[Eubacterium]_coprostanoligenes_group | 0.022993323 | 0.020535083 | 0.028279081 | 0.025040241 | 0.027859523 |
| Alistipes | 0.021469111 | 0.025946894 | 0.025388338 | 0.021773153 | 0.025399614 |
| Others | 0.552082391 | 0.432421664 | 0.514560686 | 0.541849013 | 0.44058623 |
| Unknown | 0.000553657 | 0.0000139 | 0.0008726 | 0.0000255 | 0.0000227 |
