# Supplementary material for: Effects of fermented Artemisia annua on the intestinal microbiota and metabolites of Hu lambs with naturally infected with Eimeria spp
Source: Front Cell Infect Microbiol. 2025 Jan 7;14:1448516. doi: 10.3389/fcimb.2024.1448516 (PMC11747653; doi:10.3389/fcimb.2024.1448516)
Supplement: Supplementary file 2 [file Table2.docx]

Table S2 Statistical analysis of sequencing sequences

| Sample ID | Raw Reads | Clean Reads | Denoised Reads | Merged Reads | Non-chimeric Reads |
| --- | --- | --- | --- | --- | --- |
| FA1 | 79976 | 71723 | 71119 | 57612 | 49056 |
| FA2 | 79842 | 72044 | 71643 | 61643 | 52157 |
| FA3 | 79959 | 71864 | 71364 | 59142 | 50399 |
| FA4 | 160045 | 141204 | 139962 | 121282 | 109709 |
| FA5 | 139570 | 123748 | 122382 | 102382 | 95035 |
| FA6 | 159951 | 138196 | 137158 | 119925 | 101934 |
| AA1 | 80169 | 71842 | 71361 | 58429 | 47480 |
| AA2 | 80129 | 72021 | 71620 | 60932 | 49547 |
| AA3 | 80068 | 72343 | 71749 | 59294 | 50461 |
| AA4 | 60563 | 54266 | 53892 | 46381 | 41425 |
| AA5 | 79986 | 72357 | 71951 | 61303 | 53737 |
| AA6 | 79893 | 71636 | 71035 | 59193 | 48385 |
| PL1 | 159898 | 137019 | 135901 | 113406 | 90683 |
| PL2 | 159974 | 138939 | 137955 | 119384 | 102461 |
| PL3 | 160242 | 139678 | 138728 | 123474 | 105146 |
| PL4 | 160032 | 137803 | 136959 | 118625 | 102940 |
| PL5 | 159924 | 137699 | 136863 | 118932 | 102044 |
| PL6 | 160044 | 137172 | 135939 | 116948 | 97241 |
| DI1 | 79995 | 71527 | 71035 | 61687 | 52940 |
| DI2 | 80005 | 71486 | 71068 | 62951 | 56301 |
| DI3 | 79771 | 71166 | 70649 | 60700 | 53212 |
| DI4 | 68844 | 61664 | 61349 | 55193 | 48323 |
| DI5 | 80102 | 71580 | 71018 | 58206 | 47963 |
| DI6 | 80066 | 72086 | 71724 | 64670 | 57862 |
| CON1 | 79929 | 72234 | 71789 | 61307 | 54130 |
| CON2 | 80013 | 71481 | 71080 | 62827 | 55775 |
| CON3 | 79950 | 72012 | 71522 | 61448 | 51892 |
| CON4 | 79898 | 71342 | 70789 | 61004 | 51971 |
| CON5 | 79832 | 71481 | 71040 | 58644 | 50706 |
| CON6 | 76573 | 68723 | 68271 | 56186 | 46706 |

Fermented *Artemisia annua =* FA; *Artemisia annua* = AA; Probiotic liquid = PL; Diclazuril = DI; Control = CON.
