# Supplementary material for: Effects of fermented Artemisia annua on the intestinal microbiota and metabolites of Hu lambs with naturally infected with Eimeria spp
Source: Front Cell Infect Microbiol. 2025 Jan 7;14:1448516. doi: 10.3389/fcimb.2024.1448516 (PMC11747653; doi:10.3389/fcimb.2024.1448516)
Supplement: Supplementary file 3 [file Table3.docx]

Table S3 The proportion of bacteria in each group was top 10 at phylum level

| phylum | FA | AA | PL | DI | CON |
| --- | --- | --- | --- | --- | --- |
| Firmicutes | 0.500245947 | 0.586304863 | 0.51166152 | 0.477734394 | 0.56240196 |
| Bacteroidota | 0.269875968 | 0.267035769 | 0.284635431 | 0.295293799 | 0.281460259 |
| Proteobacteria | 0.056221586 | 0.049329933 | 0.022590247 | 0.122861651 | 0.064891038 |
| Actinobacteriota | 0.016616338 | 0.035498981 | 0.022650892 | 0.04075414 | 0.012241207 |
| Verrucomicrobiota | 0.025686612 | 0.021387391 | 0.046518053 | 0.016466126 | 0.013748266 |
| Spirochaetota | 0.010153326 | 0.011741758 | 0.049403742 | 0.017317163 | 0.024816236 |
| Desulfobacterota | 0.023696975 | 0.013425586 | 0.009869967 | 0.010626803 | 0.012153701 |
| Patescibacteria | 0.022497017 | 0.005501892 | 0.012652897 | 0.006371619 | 0.008731218 |
| Campylobacterota | 0.011240788 | 0.001181452 | 0.00649238 | 0.002919661 | 0.009962793 |
| Cyanobacteria | 0.005598336 | 0.00474313 | 0.005060485 | 0.006722234 | 0.006446323 |
| Others | 0.05761345 | 0.003835387 | 0.027591772 | 0.002906912 | 0.003124311 |
| Unknown | 0.000553657 | 0.000013859 | 0.000872614 | 0.000025499 | 0.000022687 |

Fermented *Artemisia annua =* FA; *Artemisia annua* = AA; Probiotic liquid = PL; Diclazuril = DI; Control = CON.
