# Supplementary material for: Effects of fermented Artemisia annua on the intestinal microbiota and metabolites of Hu lambs with naturally infected with Eimeria spp
Source: Front Cell Infect Microbiol. 2025 Jan 7;14:1448516. doi: 10.3389/fcimb.2024.1448516 (PMC11747653; doi:10.3389/fcimb.2024.1448516)
Supplement: Supplementary file 4 [file Table4.docx]

Table S4 The proportion of bacteria in each group was top 10 at genus level

| genus | FA | AA | PL | DI | CON |
| --- | --- | --- | --- | --- | --- |
| Bacteroides | 0.093120311 | 0.099335477 | 0.06979561 | 0.092944683 | 0.146965788 |
| Christensenellaceae_R_7_group | 0.072090156 | 0.096615713 | 0.068917942 | 0.057966118 | 0.08200993 |
| Rikenellaceae_RC9_gut_group | 0.040174347 | 0.064695733 | 0.103022309 | 0.075974947 | 0.049616267 |
| UCG_005 | 0.060844956 | 0.073187633 | 0.062137499 | 0.054303791 | 0.060133269 |
| unclassified_Lachnospiraceae | 0.049822543 | 0.067394709 | 0.069015648 | 0.04900314 | 0.062414924 |
| unclassified_Muribaculaceae | 0.038623665 | 0.043540474 | 0.031183301 | 0.036489394 | 0.015530809 |
| unclassified_Clostridia_UCG_014 | 0.023218315 | 0.03717588 | 0.024997515 | 0.029480294 | 0.032335325 |
| Escherichia_Shigella | 0.025007224 | 0.039136882 | 0.001829456 | 0.015149728 | 0.057125634 |
| unclassified_[Eubacterium]_coprostanoligenes_group | 0.022993323 | 0.020535083 | 0.028279081 | 0.025040241 | 0.027859523 |
| Alistipes | 0.021469111 | 0.025946894 | 0.025388338 | 0.021773153 | 0.025399614 |
| Others | 0.552082391 | 0.432421664 | 0.514560686 | 0.541849013 | 0.44058623 |
| Unknown | 0.000553657 | 0.0000139 | 0.0008726 | 0.0000255 | 0.0000227 |

Fermented *Artemisia annua =* FA; *Artemisia annua* = AA; Probiotic liquid = PL; Diclazuril = DI; Control = CON.
